# Neotropical Rattlesnake (*Crotalus simus*) Venom Pharmacokinetics in Lymph and Blood Using an Ovine Model

**DOI:** 10.3390/toxins12070455

**Published:** 2020-07-17

**Authors:** Edgar Neri-Castro, Melisa Bénard-Valle, Dayanira Paniagua, Leslie V. Boyer, Lourival D. Possani, Fernando López-Casillas, Alejandro Olvera, Camilo Romero, Fernando Zamudio, Alejandro Alagón

**Affiliations:** 1 Departamento de Medicina Molecular y Bioprocesos, Instituto de Biotecnologia, Universidad Nacional Autónoma de México, Av. Universidad 2001, Cuernavaca 62210, Mexico; neri@ibt.unam.mx (E.N.-C.); mel@ibt.unam.mx (M.B.-V.); possani@ibt.unam.mx (L.D.P.); aolvera@ibt.unam.mx (A.O.); zam@ibt.unam.mx (F.Z.); 2 Programa de Doctorado en Ciencias Biomédicas, Universidad Nacional Autónoma de México, Unidad de Posgrado, Edificio B Primer Piso, Ciudad Universitaria, Ciudad de México 04510, Mexico; 3 Facultad de Ingeniería, Arquitectura y Diseño, Universidad Autónoma de Baja California, Ensenada, Baja California 22860, Mexico; dashpame@gmail.com; 4 Venom Immunochemistry, Pharmacology, and Emergency Response (VIPER) Institute, University of Arizona,1501 N. Campbell Avenue, Tucson, AZ 85724, USA; leslievboyer@gmail.com; 5 Instituto de Fisiología Celular, Universidad Nacional Autónoma de México, Ciudad Universitaria, Ciudad de México 04510, Mexico; fcasilla@ifc.unam.mx; 6 Centro Universitario UAEM Amecameca, Universidad Autónoma del Estado de México, Amecameca de Juárez 56900, Mexico; mvzcamilo@yahoo.com.mx

**Keywords:** *Crotalus simus* venom, differential absorption of venom protein families, lymphatic system, crotoxin, pharmacokinetics of venom

## Abstract

The most abundant protein families in viper venoms are Snake Venom Metalloproteases (SVMPs), Snake Venom Serine Proteases (SVSPs) and Phospholipases (PLA_2_s). These are primarily responsible for the pathophysiology caused by the bite of pit-vipers; however, there are few studies that analyze the pharmacokinetics (PK) of whole venom (WV) and its protein families. We studied the pathophysiology, PK profile and differential absorption of representative toxins from venom of Neotropical Rattlesnake (*Crotalus simus*) in a large animal model (ovine). Toxins studied included crotoxin (the main lethal component), which causes moderate to severe neurotoxicity; SVSPs, which deplete fibrinogen; and SVMPs, which cause local tissue damage and local and systemic hemorrhage. We found that Whole Venom (WV) was highly bioavailable (86%) 60 h following intramuscular (IM) injection, and extrapolation suggests that bioavailability may be as high as 92%. PK profiles of individual toxins were consistent with their physicochemical properties and expected clinical effects. Lymph cannulated animals absorbed 1.9% of WV through lymph during the first 12 h. Crotoxin was minimally detectable in serum after intravenous (IV) injection; however, following IM injection it was detected in lymph but not in blood. This suggests that crotoxin is quickly released from the blood toward its tissue targets.

## 1. Introduction

Forty two of the 53 species of rattlesnakes in the genus *Crotalus* are indigenous to Mexico [1]. Of these, the neotropical rattlesnake *Crotalus simus* stands out for its wide distribution. *C*. *simus* is found in the Mexican states of Veracruz, Tabasco, Oaxaca, Chiapas and in Central America as far south as Costa Rica. It has a mean length of 130 cm and is recognized as a species of medical importance [2,3]. Its venom is used in the hyperimmunization of horses to produce antivenom in Mexico [4]. The organisms formerly classified as *C. simus* that are distributed in the state of Veracruz have recently been proposed to be a new species (*C. mictlantecuhtli*) [5], but for the purpose of consistency with prior reports we will refer to them here using the traditional taxonomy.

The composition of *C. simus* venom from Mexico, Guatemala and Costa Rica has been characterized biologically and biochemically [4,6,7,8,9]. Additionally, in Mexico, the transcriptomic profile of the venom glands and proteomic profile of the venoms of juvenile and adult specimens have been documented [7]. The proteome sampled from adults has been described as consisting of 22% phospholipases type A_2_ (PLA_2_s, including crotoxin at 14%), 30% snake venom serine proteases (SVSPs), 28% snake venom metalloproteases (SVMPs), 17% other less abundant proteins and 3% non-identified proteins [4]. The venom has procoagulant activity in vitro, attributable mainly to thrombin-like enzymes, with a minimum procoagulant dose in human plasma (MPD) of 26 µg. It has a minimum hemorrhagic dose (MHD) of 37 µg in mice, attributable largely to SVMPs, and a high lethal activity with a median lethal dose (LD_50_) of 0.21 µg/g of mouse weight [4]. Lethality of the whole venom is primarily attributable to crotoxin, which is a potent neurotoxin comprised of two subunits [4,7,10]. The acidic subunit, crotoxin A or crotapotin, has a molecular weight of approximately 9.4 kDa, has no enzymatic activity, and is not toxic. The basic subunit, crotoxin B, has a molecular weight (MW) of 14.4 kDa, does have phospholipase enzymatic activity, and has toxicity on its own. When the two subunits bind and form a heterodimer, their lethality in mice increases dramatically [11,12,13,14,15].

Venom composition varies between populations found in the states of Veracruz and Chiapas [4], with little individual variation among adult *C. simus* within either range [4,7]. Unfortunately, in Mexico there are no clinical reports involving *C. simus* envenomation in Veracruz, so studies with animal models are of great importance.

Given their different MW and tissue targets, the several protein families in viper venoms are likely to have different pharmacokinetic (PK) profiles, including differences in lymphatic uptake vs. direct absorption via blood capillaries. Differences in uptake and distribution may in turn have a direct impact on the evolution of envenomation, with implications for defense, prey immobilization and efficiency of prey digestion.

One of the lymphatic system’s primary roles is the absorption of high molecular weight molecules. Supersaxo et al. [16] reported a linear relationship between the absorption of molecules and their MW (0.2 to 19 kDa). Proteins with MW greater than 16,000 are absorbed mainly by the lymphatics that drain the site of exposure. Viper venom components range in MW from 1 to 110 kDa, therefore, on this basis alone it is likely that venom toxins exhibit a range of lymphatic and blood capillary uptake characteristics.

Venom toxins, on the other hand, are not chemically inert. Enzymes such as SVMPs act preferentially in the region of the inoculation site, causing tissue damage such as dermal necrosis and local hemorrhage [17,18,19]. Both the binding to target molecules and the disruption of normal subcutaneous architecture can affect the absorption of venom. Therefore, it is important to perform PK studies both in lymph and blood, in an animal model, in order to fully understand how and when the various molecules reach their target sites following snakebite. In human and veterinary medical care, pharmacokinetic differences may explain poorly understood aspects of the natural history of envenomation, and they may inform the timing, choice and dosage of treatment with antivenom.

Blood absorption of viper venom has been described in human cases, but without formal PK analysis [20,21,22]. Animal studies following injection confirm the partial uptake of whole venom (WV) and isolated components into blood and retention in local tissue [23,24,25,26]. A review by Sanhajariya et al. showed that between 1946 and 2018 only nine formal PK studies of snake venom uptake and distribution were conducted, and in most cases these involved the venoms of elapid snakes rather than those of vipers [27]. Among these, a single study with an elapid involved the simultaneous measurement of WV and its component toxins. In this study, Yap et al. [28] studied the PK of the WV of *Naja sumatrana* and in parallel the PK of a cardiotoxin (isolated and in the context of WV) in rabbits. They described that the WV and the cardiotoxin reached their highest concentrations at 60 min and 30 min, respectively. The bioavailability (F) was 41.9% for WV and 39.5% for the cardiotoxin, suggesting that much of the venom and toxin remained at the injection site, even after 24 h. In the same study, the PK of the three main toxins of the venom (PLA_2_, α-neurotoxins and cardiotoxin) were assessed. When they were injected independently, the bioavailability (F) values were 68%, 81.5% and 45.6%, respectively. Overall, this suggested that α-neurotoxin is the predominant toxin involved in systemic envenomation by *N. sumatrana*, while the other two remain at the injection site generating local damage. Another study of an elapid venom (*Micrurus fulvius,* the Florida coral snake) in sheep demonstrated the importance of the lymphatic system in maintaining stable levels of venom in blood. In this study, an F of 60% was reported and 25% of the total absorbed venom dose was absorbed by the lymph during the first 6 h following subcutaneous injection of WV [29].

A PK analysis of WV from the viper *Hypnale* has been performed in rabbits, showing that detection of the venom is rapid following intramuscular injection, with a maximum concentration occurring before 30 min. Of particular interest in this study was the observation of three peaks of absorption during the first three hours of injection, suggesting the possibility of different toxins being absorbed at different times. The F was very low (4%), consistent with retention of most venom at the injection site [26]. Individual venom components were not measured in this study, however, leaving this interpretation open to question.

Incomplete understanding of venom toxin uptake and distribution kinetics has had direct implications for physicians involved in the study and care of patients envenomated by pit vipers. This problem has led to some disagreement related to appropriate dosing and administration of antivenom, which is available in formulations with varied target specificity and with several different pharmacokinetic profiles [21,30,31]. A number of clinical studies used enzyme-linked immunosorbent assay (ELISA) assays of serum for WV levels, but correlation of these levels with clinical effects has been generally limited to their association with hypofibrinogenemia or thrombocytopenia, rather than to local tissue injury or neurotoxicity [30]. Validated clinical study endpoints that involve non-coagulopathic effects have been limited to a composite Snakebite Severity Score [32] and the assessment of functional outcomes following copperhead snakebites [33], neither of which has been correlated with the detection or quantitation of venom toxins. Furthermore, the interpretation of whole venom levels has occasionally been called into question [34] owing to lack of certainty that specific toxins are proportionally represented in blood during the acute and subacute phases of envenomation. In addition, those studies that show a pattern of coagulopathy concurrent with venonemia have been disproportionately representative of envenomation by *C. atrox* in the southwestern USA, the distinctive coagulation profile of which may not be representative of pit vipers in general.

**Figure 1:**
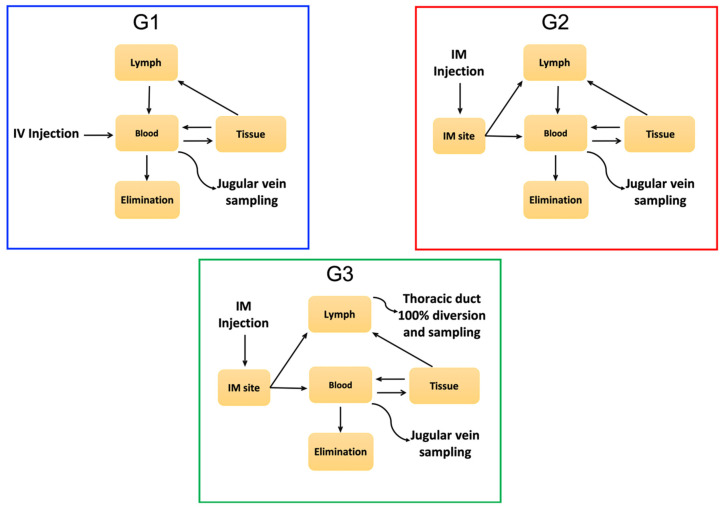
Schematic representation of experimental design to analyze the absorption, distribution and elimination of venom and the main toxin families. Animals in G1 underwent intravenous injection and sampling, for traditional pharmacokinetic analysis. 100% of venom enters the blood circulation. Animals in G2 underwent intramuscular injections and venous sampling. Slower, incomplete uptake of venom via lymph and blood changes the pharmacokinetic profile. Animals in G3 were similar to G2 but had lymph diverted from the thoracic duct, preventing its contribution to circulating blood. Whole venom (WV) and components in blood and lymph were measured quantitatively.

The purpose of this study is to report the simultaneous WV and component toxin PK of *C. simus* venom in an ovine model involving both the blood and the lymphatic systems (Figure 1), as a contribution to the improved understanding of viper venom pathophysiology.

## 2. Results

### 2.1. Venom Characterization

The pooled *C. simus* venom used in this work had an reversed-phase high-performance liquid chromatography (RP-HPLC) electrophoretic profile (Figure S1) identical to the one reported previously [4]. The mouse LD_50_ of the venom was 0.16 µg/g. Mice showed signs of neurotoxicity and had hind limb flaccid paralysis 30 min after IV venom administration with doses close to the LD_50_.

### 2.2. Size-exclusion Chromatography and Toxicity of Protein Fractions

**Figure 2:**
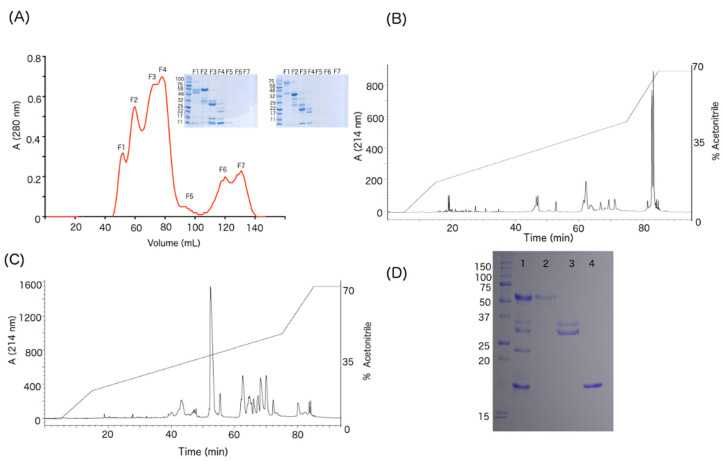
(**A**) Size-exclusion chromatography of the venom (29 mg) of *C. simus*. Seven fractions were obtained and analyzed by SDS-PAGE under reducing (left gel) and non-reducing (right gel) conditions. (**B**) Fraction 2 was analyzed by RP-HPLC showing as main components the fractions that elute after 80 min, which correspond to SVMPs-III. (**C**) Fraction 3 contained subunit B of crotoxin (red dot) and SVSPs (blue dot). (**D**) SDS-PAGE: lane 1, WV (20 µg); lane 2, SVMPs-III (9 µg); lane 3, SVSPs (9 µg); lane 4, subunit B of crotoxin (9 µg).

The WV was separated by size-exclusion chromatography (Figure 2A), yielding seven fractions. Subsequent SDS-PAGE showed protein bands for the first four fractions only. As shown in the inset of Figure 2A, FI has high molecular weight bands of approximately 50 and 110 kDa and FII has a predominant band of 55 kDa and other bands close to 32 kDa. FIII has an abundant band at 28 kDa and others around 14 and 10 kDa, while FIV shows three main bands of 22, 14 and 10 kDa.

For FI to FIV protein fractions, LD_50_, Minimum Coagulant Dose (MPD-P) and MHD results are summarized in Table 1. FI was non-lethal and lacked activity in both the MCD-P and MHD assays. FII, FIII and FIV had an LD_50_ 11.6, 0.5 and 0.1 µg/g of mouse weight, respectively. The MHD could be determined only in FII, with 10.4 µg; FII and FIII did not present MHD activity with amounts lower than 50 µg, and greater amounts could not be evaluated because the mice died from flaccid paralysis. FII, FIII and FIV presented MCD-P activity of 29, 15.8 and 50 µg, respectively (Table 1).

### 2.3. Purification of SVMPs, SVSPs, Crotoxin and Their Respective Antibodies

The FII from size-exclusion chromatography was separated by RP-HPLC, yielding two main fractions (Figure 2B). These correspond to isoforms of SVMP-III, as confirmed by partial sequences obtained in previous studies [4,7]. Similarly, subunit B of crotoxin and two isoforms of SVSPs were obtained from FIII. Crotoxin subunit B was confirmed based on a mass of 14,186 Da and neurotoxic activity when inoculated in mice with 0.5 µg/g body weight. The SVSPs were identified by N-terminal sequencing, which on protein band of ~35 kDa was VVGGHPCNINEHRSLVVLF, while that of ~30 kDa was VVGDECNINEHRSLVAIF (Figure 2C). Figure 2D shows an SDS-PAGE with the purified proteins. Subsequently, these purified proteins were used for affinity purification of antibodies as described in the methodology. Figure S2 reports the western blot results, demonstrating that the immunopurified antibodies (Section 5.12) did not have crossed recognition against other protein families. The anti-SVMPs recognized the two bands of the WV corresponding to SVMP-II and SVMP-III, whereas anti-SVSP antibodies recognized the five protein bands in WV previously described as SVSPs [4,7]. Finally, the monoclonal antibody recognized only the band of 14 kDa corresponding to the subunit B of crotoxin, consistent with previous studies [10,35].

### 2.4. Coagulation of Whole Blood

**Figure 3:**
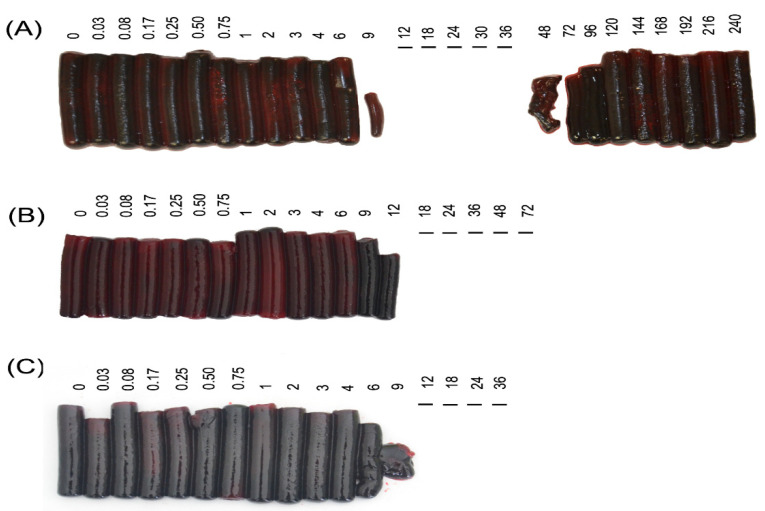
Group 2 (injected with the WV IM., blood but not lymph collected) coagulation tests. Clots come from blood samples collected at different times (shown in h), where time zero refers to the blood sample collected before administration of venom. At times where no clot is depicted this is because the clot failed to form, and the blood was completely liquid. (**A**) Sheep 2A; (**B**) Sheep 2B; (**C**) Sheep 2C.

**Figure 4:**
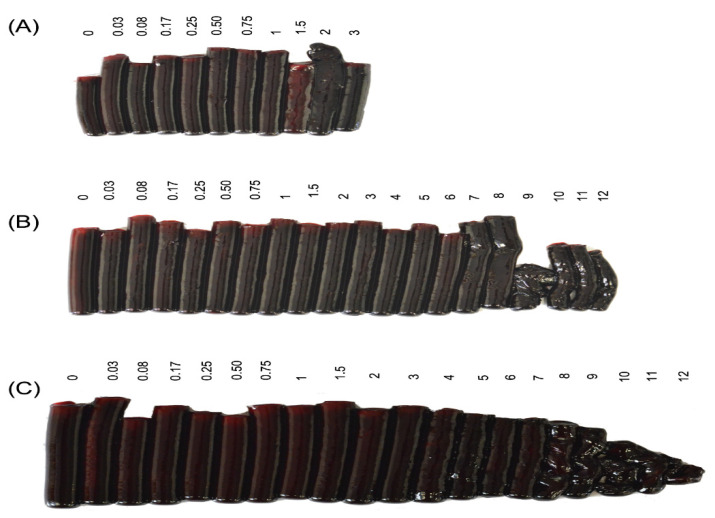
G3 (injected with the WV IM, both blood and lymph were collected) coagulation tests. The clots come from blood samples obtained at different times (shown in h) of the experiment; time zero refers to the blood sample collected before venom administration the numbers. (**A**) Sheep 3A; (**B**) Sheep 3B; (**C**) Sheep 3C.

Blood samples were collected at defined intervals from three groups of sheep (Figure 1 and Table 2). Animals in G2, which received venom injected IM, developed severe coagulation abnormalities. This is graphically illustrated in Figure 3, in which the clots produced at each time point have been removed from the red cap tubes and laid out in sequence. Sheep 2A developed clotting abnormalities 9 h after administration of venom, with complete absence of clotting between 12 and 36 h and recovery between 48 and 120 h. For the sheep 2B coagulopathy began at 12 h with a decrease in clot size, whereas no clotting was observed in samples from 18 to 72 h. Finally, for sheep 2C the coagulation alterations began at 9 h when a very small clot was observed, and from 12 h to death at 36 h no clot was formed (Figure 3). In Group 3, animal 3A did not develop coagulation alterations during the 3 h before death, whereas sheep 3B and 3C began to show abnormal clots at 9 and 8 h, respectively. These clots diminished in size and thickness until the conclusion of the experiment at 12 h, as illustrated in Figure 4B,C.

### 2.5. Fibrinogen Degradation and Quantification

**Figure 5:**
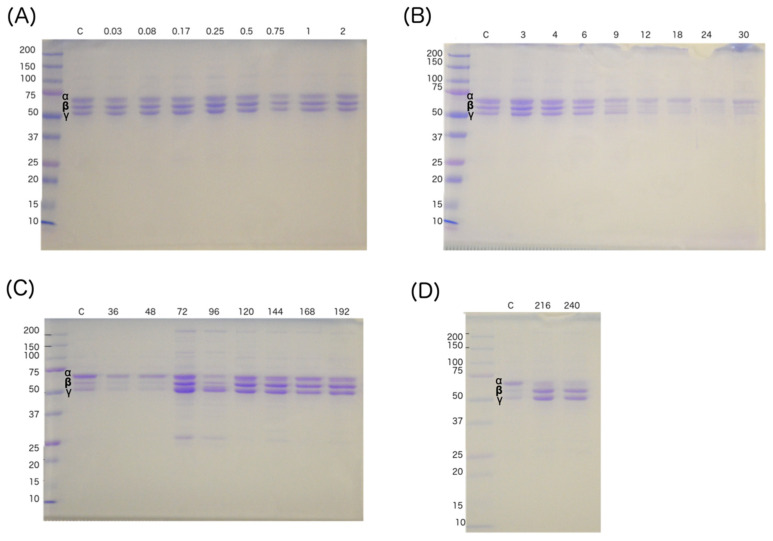
Representative fibrinogen analysis, showing results from G2 (sheep 2A). SDS-PAGE at 12.5% in the presence of 2-mercaptoethanol. The numbers above each gel indicate the time the samples were collected. (**A**) samples collected from 0.03 to 2 h; (**B**) samples collected from 3 to 30 h; (**C**) samples collected from 36 to 192 h; (**D**) samples collected at 216 and 240 h. At 12 h degradation of the fibrinogen chain α and β is observed, and at 72 h recovery is observed. Amount of fibrinogen analyzed at 9, 12, 18, 24, 30, 36 and 48 h, was 2.9, 1.2, 2.0, 1.6, 1.3, 2.0 and 3 µg, respectively. For the rest of the samples, 5 µg were analyzed. C: fibrinogen control. Some in vitro degradation is apparent in the lower two gels, attributable to a 3-h difference in timing of the assay.

Figure 5 shows a representative set of G2 gels, from sheep 2A. A decrease in the intensity of chain β is apparent starting at 6 h and continuing until 72 h, with recovery after 96 h and lasting until the end of the experiment. In the group where lymph was drained (G3), the sheep that died at 3 h (3A) did not show a decrease in the bands corresponding to fibrinogen, while animals 3B and 3C did show a decrement, starting 9 h after the inoculation of the venom. The quantifications of baseline fibrinogen of sheep G1, G2 and G3 were between 1.3 and 3.5 mg/mL. In G2 animals the concentrations of fibrinogen decreased significantly at 4 h; the concentration was 0.4 mg/mL until reaching values of 0.1 mg/mL at 12 h. In the case of the surviving sheep (2A) a recovery of the levels was observed at 72 h, when the values were similar to the baseline values (Table S1). In the case of the cannulated sheep (G3), the animal 3A (experiment that lasted 3 h) did not manifest a significant decrement of fibrinogen levels, while 3B and 3C presented a decrease at 9 h when the values were 0.7 and 1.5 mg/mL, and at 12 h presented values of 0.2 and 0.9 mg/mL, respectively (Table S2). It is important to note that the decrease in fibrinogen concentrations in G3 occurred hours later (~6 h) than in G2.

**Figure 6:**
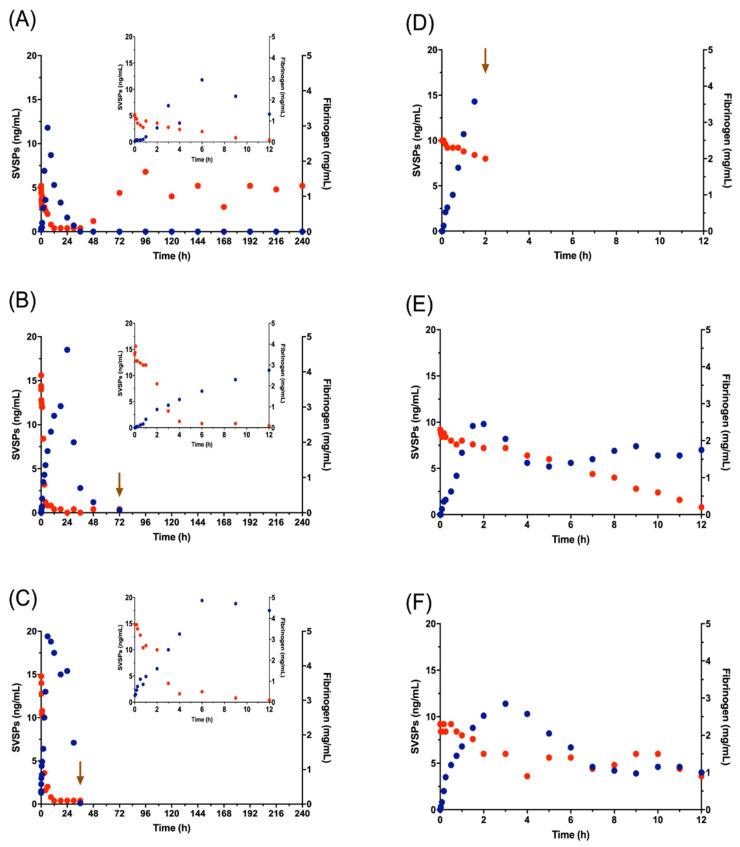
Concentrations of Snake Venom Serine Proteases (SVSPs) (blue) and fibrinogen (red) in blood. **A**, **B** and **C** show the values obtained for the three animals of G2 and **D**, **E**, and **F** show the values of G3. Inset graphs for G2 animals illustrate only the first 12 h, to facilitate comparison with G3. The arrow shows the time of death.

Figure 6 illustrates the change in fibrinogen and SVSPs concentrations that occurred over time, in G2 and G3. Beginning immediately after venom injection, all animals show a drop in fibrinogen, which continues for at least 12 h. In sheep 2A, the SVSPs fell to zero by 36 h and fibrinogen began to recover at 48 h, reaching normal concentrations.

### 2.6. Antibodies and Their Recognition

Anti-SVMP and anti-SVSP antibodies showed no cross recognition. Purified proteins and WV were analyzed by western blot, which showed that anti-SVMP antibodies recognized the purified SVMP-PIII as well as SVMP-PII and SVMP-PIII from the WV, without recognition of SVSPs or PLA_2_. The anti-SVSPs recognized the two purified SVSPs, as well as the SVSPs in WV. Absence of significant cross recognition was further confirmed by ELISA (Figure S3).

### 2.7. Pharmacokinetic Parameters of WV, SVMPs, SVSPs and Crotoxin

Measured values showed a high standard deviation (SD) attributable to the size of the sample (*n* = 3) and the intrinsic physiological variability, which has also been observed in other studies [29,36]. The quantifications of WV, SVMPs, SVSPs and crotoxin for each group are provided in Tables S3–S6. PK parameters of G1, G2 and G3 are summarized in Table 3, Table 4 and Table 5 respectively. In G3, where data were collected for only 12 h (Table 5 and Table 6), the concentration of WV and/or specific components remained constant at the end of the experiment, making it impossible to extrapolate values to infinity. Given these limitations we based our comparative analysis on the PK profile, bioavailability (F) and area under the curve (AUC _0-t_).

**Figure 7:**
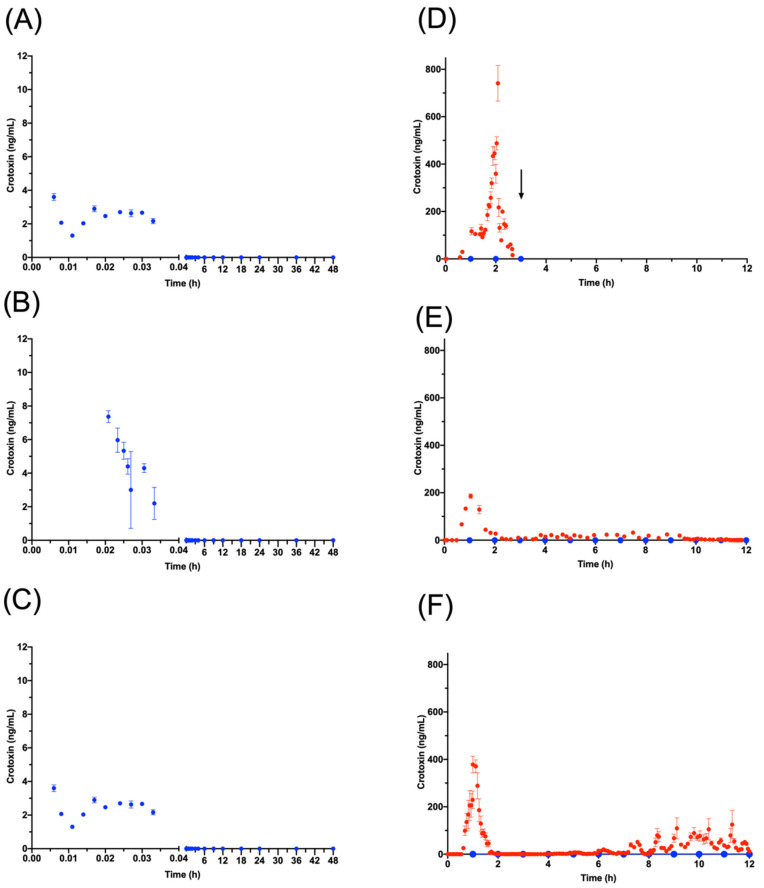
Crotoxin concentrations. In the three G1 sheep (**A**–**C**), crotoxin was quantifiable for only 2 min in blood (blue) before becoming undetectable. (**D**–**F**) show crotoxin concentrations in G3 sheep (G3A (D); G3B (E); G3C (F). In lymph (red), crotoxin could be quantified from 0.5 h to 12 h, the duration of the experiment. In the blood samples from the same animals (blue) crotoxin was undetectable throughout the experiment. The arrow in D indicates the time of death of the animal.

The PK profiles of WV, SVMPs and SVSPs were similar following IV venom injection, while crotoxin was detected only during the first two minutes (Figure 7 and Table S3d to S3f. The bioavailability of WV injected IM was 85% after 60.00 (±21.00) h, and when extrapolated to infinity it reaches 92% (G2 in Table 4). When the absorption by the lymphatic system was analyzed (G3) (Table 5), the PK was tracked for just 12 h due to technical limitations of surgery and general anesthesia. During this period, the levels in serum reached the steady state; the total bioavailability was 32%, and the fraction absorbed by the lymphatic system was 2% of the dose (Table 5). When PK parameters of G2 were analyzed during the first 12 h and compared with the PK parameters from G3 (Table 6), no differences were observed. The absorption profile through lymph showed that the absorption process was in the exponential phase and did not reach the steady state, suggesting that steady state occurs after 12 h (Table S5).

After the venom was injected IM (G2 and G3), BSA detection in blood remained low 5 min after injection (<2 ng/mL), confirming the route as intramuscular. In these animals, the SVMPs reached a bioavailability (F) of 51% of the dose after 60 (± 21.00) h, and when extrapolated to infinity this reaches 72% (Table 4). The SVSPs reached a total bioavailability (111 ± 45%) after 60 (±21) h and crotoxin was undetectable in serum during the entire experiment after IM injection. When the lymphatic absorption was analyzed after IM injection (G3), levels of crotoxin were detected in all lymph samples showing a recovery of 2.4 ± 1.7% of the dose, while the percentage of dose of the other components absorbed by lymph was 1.9 ± 0.8% of WV, 0.4 ± 0.3% of SVMPs and 1.5 ± 0.6% of SVSPs. The amount of toxins absorbed by lymph did not show a significant difference among experimental animals.

The relative abundance of SVMPs, SVSPs and crotoxin in the venom, as determined by ELISA, was 26%, 37% and 20%, respectively, close to the values reported in proteomic studies of adult specimens [4,7]. When these values were compared with the relative abundance of the protein families after injection (which were calculated using AUC_0-t toxin family /_ AUC_0-t WV_ as described in the methods), the results showed striking differences. Crotoxin was not detected in serum when the venom was injected IM (G2 and G3) and was barely detected when injected IV (for the first two minutes only, in G1), but the proportion of crotoxin in lymph was the same as that found naturally in the WV (G3). The proportion of SVMPs in plasma was similar to that in venom, after both IV and IM injection in non-lymph cannulated animals (G1 and G2), but when the lymph was drained (G3) the proportion of SVMPs declined significantly over time, in both serum and lymph. In contrast, the relative abundance of SVSPs increased in serum after IV and IM (G1 and G2) but not in serum or lymph from G3.

## 3. Discussion

### 3.1. Purification of Protein Families and Antibodies

Purification of SVMPs, SVSPs and the basic subunit of crotoxin by RP-HPLC was based on previous work performed by our group, which included partial and complete sequencing of the enzymes [4,7]. In this study, N-terminal sequences were determined in order to verify the identity of the SVSPs that can co-elute with PLA_2_ and molecular weights estimated by SDS-PAGE confirmed that they were SVSPs.

Rabbit antibodies against SVMPs and SVSPs showed no cross recognition, either in the western blot (Figure S2) or by ELISA (Figure S3).

### 3.2. Whole Venom

*Crotalus simus* venom used in this study had an RP-HPLC chromatographic profile similar to that previously reported [4]. The LD_50_ of the venom pool was 0.16 µg/g weight of mice, similar to that previously described for venoms from the same geographic location (between 0.18 and 0.32 µg/g [10]). The mice injected with venom showed clear evidence of neurotoxicity, consistent with the action of crotoxin.

Of the fractions obtained by size-exclusion chromatography, fraction four (FIV) showed the highest toxicity, with an LD_50_ of 0.10 µg/g. This fraction caused flaccid paralysis to injected animals. This potent lethality is due to crotoxin, which has 98 to 100% sequence identity with crotoxin from *C. d. terrificus* [7] whose reported LD_50_ is 0.08 µg/g [12]. FIII had an LD_50_ of 0.52 µg/g, and it also caused flaccid paralysis of experimental animals, in this case likely due to the presence of crotoxin traces (as indicated in Figure 2A, these two fractions elute quite close to one another, and crotoxin starts eluting at the end of FIII). In contrast, FI and FII showed LD_50_s above 10 µg/g. These are the fractions in which most SVMPs and SVSPs are eluted. SVMPs are known to have a relatively high LD_50_ (>5.2 µg/g) [37,38,39]. Procoagulant activity was detected in FII, FIII and FIV, of which FIII had the lowest MCD-P, of 15.3 µg. Finally, hemorrhagic activity was evident only in FII, which had an MHD of 10.4 µg. In summary, the lethality of the venom is due to crotoxin. However, although SVSPs and SVMPs have high LD_50_, they play a significant role in envenomation, the first causing fibrinogen consumption and the second degrading extracellular matrix of blood vessels and leading to local hemorrhage.

The bioavailability of WV in G2, over the duration of the experiment, was 85 ± 0.32%. When analyzed at a cutoff time of 12 h, for comparison with G3, bioavailability was 32 ± 0.13%. This value was essentially the same in lymph-cannulated animals, 30 ± 0.03% with an additional 2 ± 1% accounted for in lymph. Taken together, these results suggest that most venom injected in a pit viper bite reaches systemic availability through absorption from the blood vasculature, rather than via the lymphatics. This finding contrasts with the results of a similar lymph-cannulated model of subcutaneous envenomation by the elapid *Micrurus fulvius,* in which 25% of the absorbed dose of venom during the first 6 h was attributable to lymphatic uptake [29]. Differences in the two models may in part be attributed to the greater blood vascularization of muscle tissue [40]. An important difference between the two venoms, however, is their effect on the microvasculature following envenomation [40,41]. The venom of *M. fulvius*, which consists mainly of PLA_2_ (~14 kDa) and three-finger toxins (~6 kDa), has very little impact on vascular permeability, and the role of its lymphatic uptake can be readily related to the molecular size of the main components [29]. In contrast, SVMPs in the venom of *C. simus* have a rapid and direct impact on vascular integrity in the soft tissues adjacent to the site of venom inoculation. Damage to local lymphatic and blood capillaries contributes to edema, increased oncotic pressure, tissue degradation, and ongoing local hemorrhage, the net effect of which changes toxin absorption by both routes while maintaining a functional venom depot for many hours locally.

Our results are more consistent with those reported by Yap et al. (2014) using *N. sumatrana* venom, which despite being from an elapid is functionally very different from that of *M. fulvius*. The main components described in that study were cardiotoxins (43.6%) and PLA_2_ (26%). When these were injected IM, the F was 41.9% at 24 h, indicating that a large part of the venom remained at the inoculation site [28], more reminiscent of what is observed in North America with pit vipers than with elapids [29].

### 3.3. SVSPs

In the group of sheep that most closely resembled natural envenomation, G2, SVSPs became detectable in blood plasma at low concentrations (0.5 ± 0.7 ng/mL) 5 min following venom injection. SVSPs levels increased between 2 and 4 h, reaching mean concentrations of 4.2 ± 2 to 7.3 ± 5 ng/mL, continuing to rise until 6 to 24 h when they peaked at 10.8 ± 5.1 and 12.7 ± 6.3 ng/mL (Figure 6A, 6B and 6C; Table S4c). In G3, where all lymph was drained before its contents could join the systemic circulation, the concentration of SVSPs at 5 min was 0.3 ± 0.25, peaking at 2 and 3 h with 13.6 ± 6.3 and 15.5 ± 10 in the animals that survived the full 12 h, but then decreasing. This implies that the lymphatic contribution to blood SVSPs levels is meaningful. The difference between SVSPs in G2 and G3 is consistent with the greater drop in plasma fibrinogen levels in G2, where circulating SVSPs were presumably the primary cause of the drop. (Figure 6 and Table S2).

Most SVSPs present in *C. simus* venom have thrombin-like activity [4,7]. These enzymes are capable of cleaving one of the three fibrinogen chains. In addition, some SVMPs present in pit viper venoms have fibrin(ogen)olytic activity [42] Both mechanisms can cause a decrease in fibrinogen concentration by consumption, although from previous experiments we know that the most important enzymes responsible for coagulopathy are the SVSPs. In the SDS-PAGE of Figure 5, degradation of the β chain is apparent (beginning 9 to 12 h after venom injection). The very low fibrinogen associated with this degradation explains the clotting failure of blood collected at that time (see Figure 3). In G2, although fibrinogen concentrations dropped by half within the first 2 h (Table S1), clot formation was still apparent until after 8 h, when the concentrations decreased to 0.2 and 0.1 mg/mL.

In the surviving G3 animals, the decrease to critically low fibrinogen occurred over longer periods. In Sheep 3B this started at 9 h with <1 mg/mL of fibrinogen; Sheep 3C at 12 h still had 0.9 mg/mL (Table S2) and the decrease in clot integrity became apparent at 9 h without at least minimal clot formation still evident during the full 12 h of the experiment. The correlations between the SVSPs and the fibrinogen levels for both groups (G2 and G3) were inverse and significant (*p* = 0.05) (Figure 6).

In the absence of lymphatic drainage, the relative abundance of SVSPs increased over time, following both IV and IM injection (G1 and G2). This can be related to ongoing recirculation of the SVSPs and is reflected in the CL1 and CL2 rates, which appear to be higher than those for SVMPs after IV injection (Table 3). SVSPs are known to be fibrinogenolytic, as their major toxic activity, implying that their main target is located in the bloodstream. The location of the target and their relatively small molecular size, compared with that of SVMP PIII (the most abundant SVMP in *C. simus* venom) can explain our observations (Table S4).

### 3.4. SVMPs

SVMPs type II and III are high molecular weight enzymes that degrade proteins in the extracellular matrix [17,43,44]. In previous studies it has been reported that the main SVMPs of *C. simus* venom are PIII-SVMPs (19%) while PII-SVMPs represent 4.7% [4]. The SVMP of Costa Rican *C. simus* venom (CsH1) has low toxicity and is associated with damage to the basement membrane and with local hemorrhage [39]. At necropsy, G2 and G3 sheep exhibited hemorrhage at the inoculation site accompanied by edema, findings consistent with damage caused by SVMPs [44].

Plasma SVMPs showed a small decrement in their relative abundance after IV and IM injection (G1 and G2) indicating a high volume of distribution (Table 4), relative to central volume. This suggests extravascular binding, which would be consistent with SVMP’s ability to bind with collagen and/or with the platelet alpha-2/beta-1 collagen receptor. Previous studies suggest that SVMPs can block the platelet-binding site on collagen [45].

SVMP blood concentrations after IM injection in G2 and G3 approached zero during the first 60 min, with maximum concentrations of 2 ng/mL. This is consistent with retention of proteins at the inoculation site.

### 3.5. Crotoxin

In our observations, crotoxin showed an unusual absorption profile in that it was only barely detected in serum after IV injection during the first 2 min and was not detected at all in serum after IM injection. When lymph samples were analyzed in G3, however, high concentrations of crotoxin were detected during the entire 12 h following IM injection (Figure 7). This is consistent with a study carried out by Barral-Neto [46], in which the crotoxin of *C. d. terrificus* was quickly cleared in mice following injection into the foot-pad, remaining quantifiable only up to 30 min.

We performed experiments (data not shown here) in which WV containing known amounts of crotoxin was added to blood ex vivo and subsequently quantified by ELISA, and these showed that the concentration was not underestimated. This demonstrated that crotoxin does not adhere to blood cells. Our interpretation of the rapid drop in IV crotoxin is therefore that a significant percentage of crotoxin is absorbed by lymphatic system and as it reaches blood it is rapidly removed, by unknown mechanisms but ultimately binding directly to the target at the presynaptic membrane. Additional experiments are anticipated to confirm this conclusion, by labeling crotoxin prior to injection in the same sheep model.

Finally, the other components that have been detected during the relative abundance calculations were not specifically characterized in this research. Their relative abundance remains to be related to their intrinsic pharmacological activity and molecular size, and also by the different affinities of the antibodies that make up the ELISA for WV detection and quantification

### 3.6. Pharmacokinetics and Its Clinical Implications

The timing of absorption and distribution of venom is of direct clinical consequence both in the natural history of envenomation and in its management with antivenom. In this study we demonstrated that there are important differences between the pharmacokinetic profile of the venom of *C. simus* and that of *M. fulvius*, the only other venom previously studied using a similar lymphatic model. These differences are explained both by physicochemical characteristics of the molecules involved and by the greater local tissue damage caused by *C. simus* venom. Damage in the region of injection has been reported by others to result in altered lymphatic and blood vascular uptake of venom from *Pseudonaja textilis* [41] and from *Bothrops asper* [47], consistent with this concept, which alters vascular integrity in the region of injection. In addition, following injection of whole *C. simus* venom we observed differences in the blood and lymphatic uptake of the several most important toxic components. These differences are associated with differences in the timing of the pathophysiological consequences in envenomation.

In Mexico, where there have been few formal medical reports of envenomation by rattlesnakes, these studies may be of clinical value at a fundamental level to understand and predict the symptoms following snakebite in humans. Of broader interest, we have also demonstrated that the neurologically important component crotoxin leaves the blood circulation so rapidly—and is therefore made available to target tissue [46]—that it is essentially unmeasurable in serum, although its ongoing absorption by the lymphatic system is readily demonstrated using the lymph-cannulated sheep model. This implies that early administration of antivenom is important in the event of snakebite involving crotoxin-like neurotoxic components. It also indicates that it is important to monitor for ongoing evidence of envenomation, because crotoxin present in lymph has the potential to cause neurotoxicity if insufficient antivenom has been used.

The pharmacokinetic profiles of SVSPs and SVMPS are different from those of crotoxin, as described in Section 3.3 and Section 3.4, respectively. In clinical practice, an improved understanding of the differences in pharmacokinetics of venom components, and of their mechanisms of action, may enable better dosing and timing of antivenom administration.

## 4. Conclusions

The venom of *C. simus* has three particularly important components in the systemic physiopathology of envenomation in sheep: crotoxin, SVSPs and SVMPs. In envenomation by *Crotalus simus*, lymph plays a different role in absorption from that reported with the venom of *Micrurus fulvius,* with relatively minor lymph absorption overall. Local damage at the *C. simus* venom depot facilitates direct systemic absorption of the venom, via the blood vasculature. SVMPs remain active in the inoculation site, contributing to local tissue injury and hemorrhage, while SVSPs undergo greater lymphatic absorption, enabling a small but constant contribution that maintains SVSPs in blood circulation and contributes to fibrinogen degradation and aberrant clotting for at least 24 h. Similarly, although crotoxin disappears very quickly after arriving in the blood circulation, it remains quantifiable in lymph for at least 12 h, suggesting that its presence in lymph may serve to potentiate neurotoxic effects over time

## 5. Materials and Methods

### 5.1. Ethics Statement

Animal experiments were approved by the Bioethics Committee of the Instituto de Biotecnología (IBt) of the Universidad Nacional Autónoma de México (UNAM, project # 319; approved on 14 February 2017). All animals used during this project (sheep, rabbits and mice) were monitored by a veterinarian for the duration of the experiments.

### 5.2. Animals

Male Katadin sheep, ranging in weight from 52 to 69 kg, were acquired from a vendor in Venustiano Carranza, State of Puebla, Mexico. We used three New Zealand rabbits to obtain polyclonal antibodies against *C. simus* venom and against BSA. For lethality tests, we used CD1 mice weighing from 18 to 20 g. Rabbits and mice were provided by the IBt vivarium, and all animals were maintained with food and water ad libitum.

### 5.3. Venom

Equal amounts of venoms that had been collected from 18 adult *C. simus* specimens by the IBt-UNAM venom bank were pooled. The snakes involved were wild caught specimens from different locations of the State of Veracruz, Mexico. Venom was collected as previously reported [48] and stored lyophilized at 4 °C.

### 5.4. Protein Quantification

Protein concentration of venom was determined using absorbance at 280 nm with the assumption that one absorbance unit, one cm light path, was equal to 1 mg/mL. Quantification of antibodies and fibrinogen were done assuming that 1.4 and 1.5 absorbance units, respectively, were equal to 1 mg/mL.

### 5.5. SDS-PAGE

SDS-PAGEs were run in 12.5% polyacrylamide gels on a Mini-protean III system (Bio-Rad, Carlsbad, CA, USA) using the discontinuous system. Depending on the sample, between 2 and 15 µg of protein were analyzed under reducing and non-reducing conditions. Protein molecular weight markers (Biolabs and Bio-Rad) were used and gels were stained with 0.2% R-250 Coomasie brilliant blue [49].

### 5.6. Lethality

The Median Lethal Dose (LD_50_) of whole or fractionated *C. simus* venom was determined in groups of three mice [50] (CD1 strain) by injecting different amounts of venom in a final volume of 0.2 mL of PBS. Injections were administered IV via the caudal vein of three mice per concentration [50]. After 24 h, the number of deaths was recorded and the corresponding LD_50_ values were estimated by non-linear regression as previously described [51,52].

### 5.7. Procoagulant Activity

The procoagulant activities of WV and fractions were analyzed through the determination of a Minimum coagulant Dose in Plasma (MCD-P), using the method previously described [53].

### 5.8. Local Hemorrhagic Activity

The hemorrhagic activity of the venom pool and fractions were analyzed through the determination of a Median Hemorrhagic Dose (MHD), using the method of Theakston and Reid [53], as modified by Gutiérrez [54].

### 5.9. Separation and Purification of SVMPs, SVSPs, and Crotoxin from Pooled C. simus Venom

Purified toxins for production, by immuno-purification, of specific antibodies was important in order to prevent cross recognition against other protein families, particularly those of the other toxins under study. Venom dissolved in PBS was fractionated by size-exclusion chromatography on a Sephadex G-75 column (0.9 × 196 cm) at a flow rate of 13 mL/h. Samples of one mL were collected and absorbance measured at 280 nm. Pooled fractions were stored frozen at −70 °C. SVMPs, SVSPs and crotoxin were purified as previously described [4,10]. We separately ran 2 to 3 mg of the FII and FIII fractions dissolved in 500 μL of solvent A (0.1% trifluoroacetic acid; TFA) and separated them using RP-HPLC using an Agilent 1100 chromatograph and a C18 column (Vydac^®^, Deerfield, IL, USA, 218 TP 4.6 mm × 250 mm), monitored at 280 nm. Elution was performed at 1 mL/min, via a gradient toward solvent B (100% acetonitrile, containing 0.1% TFA), as follows: 0% B for 5 min, 0–15% B over 10 min, 15–45% B over 60 min, 45–70% B over 10 min, and 70% B over 9 min [55,56]. We recovered SVMPs from the FII fraction and SVSPs and crotoxin from the FIII fraction.

### 5.10. Amino Acid Sequence Determination

Amino-terminal sequencing of SVSPs was determined by automated Edman degradation on a PPSQ-31A Protein Sequencer (Shimadzu, Tokyo, Japan).

### 5.11. Production of Antibodies

Venom antibodies were generated by immunizing three rabbits with increasing amounts of pooled *C. simus* venom over time. The first immunization injection was made with 5 µg of venom emulsified in 0.6 mL of Freud’s incomplete adjuvant (IFA) (Sigma, St. Louis, MO, USA), and subsequent immunizations were done every 14 days with increasing amounts of venom (25, 50, 100, 250, 500, 1000, 2000, 4000 and 6000 µg/rabbit) with the alternate use of IFA and ALUM (Thermo Fisher, Rockford, IL, USA) as adjuvants. Every 30 days during the immunization period, blood samples (1 mL) were collected to evaluate titers. Rabbits were euthanized after titers reached 30,000. Serum from the three rabbits was combined for subsequent antibody purification. Hyperimmune anti-BSA serum was produced in three rabbits by standard immunization with >98% pure BSA (Sigma-Aldrich, St. Louis, MO, USA).

### 5.12. Antibody Purification

For purification of antibodies, 10 mg of WV was covalently bound to 2 mL of cyanogen bromide-activated Sepharose 4B (CNBr-Sepharose) beads (Sigma-Aldrich). Thirty-five mL of anti-C-simus rabbit serum was passed through an affinity column prepared with this material. Unbound proteins were removed with washing buffer (50 mM Tris-HCl pH 8, containing 0.5 M NaCl). The bound antibodies were eluted with 100 mM acetic acid and received in 1M Tris-HCl buffer at pH 8, in a ratio of 1:10 (Tris:acetic), and subsequently dialyzed against PBS. Three milligrams of these antibodies were biotinylated (see Section 5.13). Non-biotinylated antibodies were used as capture antibody in an ELISA to measure WV in blood and lymph samples.

Affinity chromatography resins for the purification of SVMP and SVSP antibodies were produced by coupling, separately, 5 mg of each protein to 1 mL of CNBr-Sepharose. The corresponding antibodies were purified using 15 mL of anti-*C.* *simus*. These were then rendered specific via negative selection; that is, SVMPs were passed through SVSPs-Sepharose 4B and SVSPs through SVMPs-Sepharose, and the unbound antibodies were used in the ELISAs (Section 5.12). Aliquots of these specific antibodies were biotinylated (Section 5.13) to be used in the ELISAs for the measurement of SVMPs or SVSPs in blood and lymph.

Anti-BSA antibodies were similarly purified via immuno-purification using a BSA-Sepharose 4B column, with subsequent negative immuno-purification using an ovine serum albumin-Sepharose 4B column.

### 5.13. Biotinylation of Antibodies

We independently biotinylated two mg of anti-*C.* *simus*, anti-SVSPs, anti-SVMPs and anti-BSA antibodies, at a concentration of 1 mg/mL with 13.2 µL of biotin at a concentration of 3.4 mg/mL, using reagents and protocol from EZ-Link NHS-Biotin Reagents (Thermo Scientific, Rockford, IL, USA). Unconjugated biotin was subsequently eliminated by dialysis.

### 5.14. Quantification of WV, SVSPs, SVMPs, Crotoxin and BSA by ELISA

For ELISAs, the solutions, reagents, and protocol previously described were used [10,35]. ELISAs to quantify WV, SVMPs, and SVSPs in serum and in lymph were performed using 96-well plates coated with 5 µg/mL of their respective capture polyclonal antibodies at 37 °C for one hour. The reaction was then blocked with gelatin at 37 °C for 2 h. Because venom and fractions varied in their concentration in blood and lymph and at different times of sampling, we tested serum and lymph at 1:2, 1:5, 1:10 and 1:20 dilutions, and used the value(s) that fit the corresponding standard curve. Subsequently, specific biotinylated polyclonal antibodies were added at a concentration of 2 µg/mL and incubated for 1 h at 37 °C. Then 100 µL/well of streptavidin coupled to HRP at a concentration of 1 µg/mL were added for 1 h at 37 °C. Finally, 100 µL of buffer containing the substrate for peroxidase (ABTS) was added and the plates were read at 405 nm using a Magellan spectrophotometer. The standard curve of the WV started at 15 µg/mL followed by 1:3 serial dilutions. For SVMPs and SVSPs we started at 2 µg/mL with 1:3 serial dilutions and incubated them for 1 h at 37 °C.

The ELISA for quantitation of the subunit B of crotoxin has been reported previously [10,35]. This method uses the monoclonal antibody 4F6, which has been demonstrated to bind crotoxin without cross reaction against other venom PLA_2_s. The presence of BSA was confirmed by a similar ELISA.

The data were analyzed using nonlinear, sigmoidal dose-response analysis (variable slope) in the program GraphPad Prism V8.0b (GraphPad Software, San Diego, CA, USA), as previously described [51].

Percentages of SVMPs, SVSPs, and crotoxin were determined in WV using specific ELISA quantification for each of the protein families.

### 5.15. Ovine Model

Three groups of three sheep each were used (Figure 1 and Table 2). Blood samples were collected in tubes containing sodium citrate as an anticoagulant (blue cap Vacutainer) and tubes without anticoagulant (red cap Vacutainer), and lymph was collected as described in Section 5.16. Group 1 (G1) was injected intravenously into the jugular vein with a sublethal dose of 0.02 mg/kg of C. simus venom in a final volume of 1 mL. In G1, serial blood samples were collected at time 0 and as quickly as possible during the first two minutes, then at 5, 10, 15, 30, and 45 min and at 1, 2, 3, 4, 6, 9, 12, 18, 24, 30, 36, and 48 h. Group 2 (G2) was injected intramuscularly into the middle of the gluteus muscle with a dose of 0.15 mg/kg of venom in a final volume of 1 mL. In G2 blood samples were collected at 0, 2, 5, 10, 15, 30, and 45 min, and at 1, 2, 3, 4, 6, 9, 12, 18, 24, 30, 36, and 48 h, then every 24 h until the 10th day or until the animal died. Group 3 (G3) animals were provided general anesthesia as described in Section 5.17, then injected intramuscularly with a dose of 0.15 mg/kg of venom. In G3, blood was collected at the same times as for G2, and in addition lymph was collected continuously for a period of 12 h or until death from envenomation. To ensure that injections were truly intramuscular, and not IV, 0.15 mg/kg bovine serum albumin (BSA) was co-injected with venom as a marker in G2 and G3. BSA followed the expected absorption to blood through the lymphatic system (data not shown).

### 5.16. Blood and Lymph Processing

Blood samples in red cap tubes were kept at room temperature for 1 h, then centrifuged at 1500× *g* for 7 min. The serum was recovered in Eppendorf tubes and stored at −18 °C pending ELISA analysis, and the clots were stored for visual comparison across all time points. Citrated plasma was separated by centrifugation and stored at −18 °C pending fibrinogen determination. Lymph samples (15 mL) were collected continuously from G3 sheep throughout the surgery in conical tubes (Falcon), obtaining all lymph volume available via the cannulated thoracic duct. Lymph was allowed to clot for one hour before centrifugation and the supernatant was collected for storage at −18 °C.

### 5.17. Surgical Technique for G3 Sheep

Before surgery, we injected 1 mL of 3% Evans Blue dye subcutaneously into all four legs of G3 sheep to facilitate visualization of the thoracic duct. Sheep were then anesthetized and treated surgically as previously described, with cannulation of the lymphatic duct such that 100% of ductal lymph was diverted for collection and analysis [29,36]. For the duration of surgery, we replaced lymph with IV saline solution at a volume matching that of lymph obtained. At the end of the experiment, sheep were sacrificed with an overdose of pentobarbital.

### 5.18. Purification and Quantification of Fibrinogen

Blood samples in 4.5 mL citrate tubes were centrifuged at 1500× *g* for 10 min, then 1.4 mL was taken from the supernatant and passed to a tube containing 0.4 mL of 50% ammonium sulfate (*w*/*v*). This was mixed and centrifuged at 14,000× *g* for 1 min, the supernatant was discarded and the pellet was washed three times with 12% ammonium sulfate (*w*/*v*). The resulting pellet was redissolved in 1.4 mL of PBS, then fibrinogen was quantified by A_280 nm_ (see Section 5.4). The correlation between fibrinogen levels and SVSP concentrations in serum was determined using a Pearson correlation coefficient.

### 5.19. Pharmacokinetic Analysis

PK parameters were determined using the Microsoft add-in tool, PKSolver [57]. Parameters obtained after IV administration were estimated using a two-compartment model for IV bolus. Parameters after IM administration were estimated using a noncompartmental analysis for extravascular administration. The analysis was performed independently for each sheep and mean and standard deviation were calculated using standard Excel formulas. The amounts of venom and venom components absorbed by lymph were calculated by multiplying the concentration obtained in each lymph sample by the corresponding volume, and the cumulative amount was determined as the sum of these quantities. Total recovery of venom was calculated as the sum of systemic availability in blood (F) and cumulative fraction of the dose recovered in lymph. ELISA results were then used to quantify the principal protein families and to compare the percentages with those found in the venom pool (SVMPs 25.6%, SVSPs 36.6% and crotoxin 20.0%).

### 5.20. Relative Abundance of the Main Venom Components

To analyze how the proportion of each toxin family changed due to differences in their PK, the proportion of each family in the WV was analyzed in the different experimental groups and expressed as percentage of the WV. For this analysis the AUC0-t of each toxin family was divided by the AUC0-t of the WV during the same experiment (AUC_0-t toxin family_/AUC_0-t WV_) and multiplied by 100 to establish it as a percentage. Relative abundance of SVMP, SVSPs and crotoxin after the PK in the three experimental groups was statistically compared with the abundance measured by ELISA prior to injection by a t-test using Graph Pad Prism v.6 (GraphPad Software, San Diego, CA, USA).

## Figures and Tables

**Table 1:** Toxicity and biological activities of the WV and fractions.

Activity	FI	FII	FIII	FIV	WV
LD_50_ (µg/g)	>11.84	11.5	0.52	0.1	0.16
		(10.6 ± 12.5)	(0.46 ± 0.57)	(0.07 ± 0.1)	(0.13 ± 0.18)
MCD-P (µg)	>100	29 ± 2	15.3 ± 1	50 ± 3.6	23 ± 1
MHD (µg)	>100	10.4 ± 1.6	>50	>50	29 ± 2

LD_50_: Median Lethal Dose, dose of venom that induces death in 50% of injected mice (18–20 g) via IV injection; values in parenthesis represent 95% confidence intervals. MCD-P: Minimum Coagulant Dose, dose of venom (μg) that induces clotting of citrated human plasma in 60 s MHD: Minimum Hemorrhagic Dose, dose of venom that induces a hemorrhagic halo of 10 mm diameter in mice (25–28 g) 3 h after intradermal venom injection.

**Table 2:** Experimental groups.

Experimental	Sheep ID	Route of Venom	Sample	Venom	Duration of Experiment
Group		Administration		Dose (mg/kg)	(h)
G1	1A, 1B, 1C	I.V.	Blood	0.02	48, 48, 48
G2	2A, 2B, 2C	I.M.	Blood	0.15 *	240, 72, 36
G3	3A, 3B, 3C	I.M.	Blood and lymph	0.15 *	3, 12, 12

Route of administration intravenous (I.V.), intramuscular (I.M.) Sheep ID refers to the identification of individual sheep in each group. * Co-injected with 0.15 mg/kg of BSA.

**Table 3:** Pharmacokinetic parameters of WV, Snake Venom Metalloproteases (SVMPs) and Snake Venom Serine Proteases (SVSPs) following intravenous injection in G1.

Parameter	WV	SVMP	SVSP
Administration route	I.V.	I.V.	I.V.
Dose (mg)	1.04	0.27	0.38 (±0.00)
tz (h)	48	48	40.00 (±6.93)
k_10_ (1/h)	1.91 (±0.44)	2.26 (±0.42)	0.81 (±0.14)
k_12_ (1/h)	3.25 (±0.36)	2.40 (±0.49)	0.69 (±0.58)
k_21_ (1/h)	1.61 (±0.46)	2.68 (±0.38)	1.27 (±1.78)
t_1/2_ α (h)	0.11 (±0.02)	0.11 (±0.02)	0.45 (±0.28)
t_1/2 β_ (h)	1.52 (±0.49)	0.75 (±0.10)	4.06 (±3.28)
C_0_ (ng/mL)	97.88 (±57.79)	21.34 (±10.72)	28.61 (±6.82)
V_1_ (l)	13.14 (±6.65)	14.40 (±5.80)	13.77 (±2.93)
CL_1_ (l/h)	25.73 (±13.81)	34.10 (±18.26)	11.24 (±3.68)
V_2_ (l)	25.47 (±7.70)	12.86 (±5.55)	16.67 (±11.93)
CL_2_ (l/h)	43.11 (±21.83)	34.19 (±15.90)	10.10 (±9.96)
AUC _0-t_ (ng × h/mL)	55.76 (±42.98)	10.17 (±7.35)	36.03 (±10.75)
AUC _0-∞_ (ng × h/mL)	55.77 (±43.00)	10.32 (±7.23)	36.19 (±10.79)
AUMC (ng × h^2^/mL)	110.10 (±116.87)	9.66 (±8.59)	112.39 (±67.40)
MRT (h)	1.69 (±0.55)	0.86 (±0.17)	2.93 (±1.47)
Vss (l)	38.61 (±13.92)	27.27 (±11.29)	30.43 (±12.36)
F_0-t_	1	1	1
F_0-∞_	1	1	1

tz, time of last analytically quantifiable concentration; k_10_, elimination rate constant; k_12_, transfer rate constant from central to peripheral compartment; k_21_, transfer rate constant from peripheral to central compartment; t_1/2_ α, distribution half-life; C_0_, initial venom concentration; t_1/2 β_, elimination half-life; V_1_,central volume of distribution; CL_1_, systemic clearance; V_2_, peripheral volume of distribution; CL_2_, rapid distribution; AUC _0-t_, area under the concentration-time curve from zero up to a definite time; AUC _0-∞_, area under the concentration-time curve from zero to infinity; AUMC, area under the first moment of the concentration-time; MRT, mean residence time; Vss, apparent volume of distribution at equilibrium determined after intravenous administration; F_0-t_, fraction of the administered dose systemically available; F_0-∞_, fraction of the administered dose systemically available. The pharmacokinetics (PK) parameters were calculated from the data from the three sheep that make up each group.

**Table 4:** Parameters of WV, SVMPs and SVSPs following intramuscular injection in G2.

Parameter	WV	SVMP	SVSP
Administration route	I.M.	I.M.	I.M.
Dose (mg)	8.80 (±0.35)	2.25 (±0.09)	3.22 (±0.13)
tz (h)	60.00 (±21.00)	60.00 (±21.00)	58.00 (±24.25)
ʎ_z_ (1/h)	0.16 (±0.16)	0.06 (±0.06)	0.08 (±0.05)
t_1/2_ (h)	8.05 (±5.63)	19.84 (±18.67)	12.09 (±7.34)
t_max_ (h)	9.00 (±3.00)	22 (±3.46)	13.00 (±9.64)
C_max_ (ng/mL)	17.36 (±4.83)	1.94 (±1.17)	16.56 (±4.17)
CL/F (l/h)	23.35 (±11.48)	43.56 (±22.55)	10.09 (±6.51)
AUC _0-t_ (ng × h/mL)	400.01 (±141.76)	43.90 (±27.24)	337.98 (±134.62)
AUC _0-∞_ (ng × h /mL)	431.24 (±166.82)	61.10 (±31.62)	408.78 (±222.47)
AUMC (ng × h^2^/mL)	8603.77 (±4878.09)	1798.63 (±1493.23)	10,041.29 (±7825.81)
MRT (h)	19.49 (±4.90)	26.70 (±10.63)	21.89 (±7.42)
Vss (l)	445.49 (±219.54)	1043.13 (±139.17)	189.60 (±498.8)
Vz/F (l)	268.15 (±208.32)	943.25 (±527.95)	134.30 (±12.58)
F_0-t_	0.85 (±0.32)	0.51 (±0.34)	1.11 (±0.45)
F_0-∞_	0.92 (±0.38)	0.72 (±0.40)	1.34 (±0.71)

tz, time of last analytically quantifiable concentration; ʎ_z_, terminal rate constant; t_1/2_ terminal half-life; t_max_, time to reach maximal concentration; C_max_, maximum concentration; CL/F, apparent total plasma or serum clearance of drug after extravascular administration; AUC _0-t_,area under the concentration-time curve from zero up to a definite time; AUC _0-∞_, area under the concentration-time curve from zero to infinite; AUMC, area under the first moment of the concentration-time; MRT, mean residence time; Vss, apparent volume of distribution at equilibrium determined after intravenous administration; Vz/F, apparent volume of distribution during terminal phase after extravascular administration; F_0-t_, fraction of the administered dose systemically available between time zero and a definite time; F_0-∞_, fraction of the administered dose systemically available between time zero and infinity. The PK parameters were calculated from the data from the three sheep that make up each group.

**Table 5:** Pharmacokinetic parameters of WV, SVMPs and SVSPs following intramuscular injection in G3.

Blood Absorption			
Parameter	WV	SVMP	SVSP
Administration route	I.M.	I.M.	I.M.
Dose (mg)	9.65 (±0.17)	2.47 (±0.44)	3.53 (±0.06)
tz (h)	8.90 (±5.37)	9.00 (±5.20)	9.00 (±5.20)
ʎ_z_ (1/h)	0.03 (±0.03)	0.05 ^c^	0.07 (±0.08)
t_1/2_ (h)	37.96 (±34.52)	14.50 ^c^	32.16 (±37.26)
t_max_ (h)	2.40 (±0.79)	5.5 (±5.68)	2.67 (±0.58)
C_max_ (ng/mL)	31.57 (±17.30)	1.42 (±0.78)	17.25 (±11.55)
CL/F (l/h)	14.19 (±11.39)	91.53 ^c^	18.13 (±18.10)
AUC _0-t_ (ng × h/mL)	156.45 (±53.29)	6.61 (±2.50)	69.23 (±15.50)
AUC _0-∞_ (ng × h/mL)	1006.90 (±823.01)	26.43 ^c^	390.17 (±393.58)
AUMC (ng × h^2^/mL)	76,644.93 (±96,707.77)	624.10 ^c^	29,250.31 (±39,833.31)
MRT (h)	55.36 (±50.80)	23.61 ^c^	47.79 (±53.88)
Vss (l)	496.26 (±90.33)	2161.29 ^c^	379.04 (±112.4)
Vz/F (l)	493.32 (±83.19)	1914.45 ^c^	354.86 (±135.38)
F_0-t_	0.30 (±0.03)	0.07 (±0.03)	0.08 (±0.02)
F_0-∞_	1.94 (±1.56)	0.28 ^c^	0.46 (±0.46)
**Lymphatic Absorption**			
Venom in lymph (mg)	0.187 (±0.08)	0.01 (±0.006)	0.05 (±0.02)
F(lymph) 0-t	0.02 (±0.01)	0.004 (±0.003)	0.01 (±0.01)
**Total Absorption**			
F total 0-t	0.32 (±0.11)	0.074 (±0.03)	0.10 (±0.02)

tz, time of last analytically quantifiable concentration; ʎ_z_, terminal rate constant; t_1/2_ terminal half-life; t_max_, time to reach maximal concentration; C_max_, maximum concentration; CL/F, apparent total plasma or serum clearance of drug after extravascular administration; AUC _0-t_,area under the concentration-time curve from zero up to a definite time; AUC _0-∞_, area under the concentration-time curve from zero to infinite; AUMC, area under the first moment of the concentration-time; MRT, mean residence time; Vss, apparent volume of distribution at equilibrium determined after intravenous administration; Vz/F, apparent volume of distribution during terminal phase after extravascular administration; F_0-t_, fraction of the administered dose systemically available between time zero and a definite time; F_0-∞_, fraction of the administered dose systemically available between time zero and infinity. The PK parameters were calculated from the data from the three sheep that make up each group.

**Table 6:** Comparison of pharmacokinetic parameters of WV, SVMPs and SVSPs in G2 and G3 at 12 h.

Blood Absorption						
Parameter	G2.WV	G3.WV	G2.SVMP	G3.SVMP	G2.SVSP	G3.SVSP
Administration route	I.M.	I.M.	I.M.	I.M.	I.M.	I.M.
Dose (mg)	8.80 (±0.35)	9.65 (±0.17)	2.25 (±0.09)	2.47 (±0.44)	3.22 (±0.13)	3.53 (±0.06)
tz (h)	12	8.90 (±5.37)	12	9.00 (±5.20)	12.00 (±0.00)	9.00 (±5.20)
ʎ_z_ (1/h)	0.02 ^c^	0.03 (±0.03)	0.005 ^c^	0.05 ^c^	ND	0.07 (±0.08)
t_1/2_ (h)	34.78 ^c^	37.96 (±34.52)	151.10 ^c^	14.50 ^c^	ND	32.16 (±37.26)
t_max_ (h)	9.00 (±3.00)	2.40 (±0.79)	9.33 (±4.62)	5.5 (±5.68)	9.00 (±3.00)	2.67 (±0.58)
C_max_ (ng/mL)	17.36 (±4.83)	31.57 (±17.30)	1.72 (±1.07)	1.42 (±0.78)	14.07 (±4.63)	17.25 (±11.55)
CL/F (l/h)	8.36 ^c^	14.19 (±11.39)	13.50 ^c^	91.53 ^c^	ND	18.13 (±18.10)
AUC _0-t_ (ng × h/mL)	151.83 (±62.13)	156.45 (±53.29)	10.75 (±2.90)	6.61 (±2.50)	115.25 (±50.63)	69.23 (±15.50)
AUC _0-∞_ (ng × h/mL)	1076.16 ^c^	1006.90 (±823.01)	170.78 ^c^	26.43 ^c^	ND	390.17 (±393.58)
AUMC (ng × h^2^/mL)	55,943.52 ^c^	76,644.93 (±96,707.77)	37,576.65 ^c^	624.10 ^c^	ND	29,250.31 (±39,833.31)
MRT (h)	51.98 ^c^	55.36 (±50.80)	220.03 ^c^	23.61 ^c^	ND	47.79 (±53.88)
Vss (l)	434.75 ^c^	496.26 (±90.33)	2968 ^c^	2161.29 ^c^	ND	379.04 (±112.4)
Vz/F (l)	419.61 ^c^	493.32 (±83.19)	2940 ^c^	1914.45 ^c^	ND	354.86 (±135.38)
F_0-t_	0.32 (±0.13)	0.30 (±0.03)	0.11 (±0.03)	0.07 (±0.03)	0.37 (±0.16)	0.08 (±0.02)
F_0-∞_	2.23 ^c^	1.94 (±1.56)	1.82 ^c^	0.28 ^c^	ND	0.46 (±0.46)
**Lymphatic Absorption**						
Venom in lymph (mg)	NA	0.187 (±0.08)	NA	0.01 (±0.006)	NA	0.05 (±0.02)
F(lymph) 0-t	NA	0.02 (±0.01)	NA	0.004 (±0.003)	NA	0.01 (±0.01)
**Total Absorption**						
F total 0-t	0.32 (±0.13)	0.32 (±0.11)	0.11 (±0.03)	0.074 (±0.03)	0.37 (±0.16)	0.10 (±0.02)

tz, time of last analytically quantifiable concentration; ʎ_z_, terminal rate constant; t_1/2_ terminal half-life; t_max_, time to reach maximal concentration; C_max_, maximum concentration; CL/F, apparent total plasma or serum clearance of drug after extravascular administration; AUC _0-t_,area under the concentration-time curve from zero up to a definite time; AUC _0-∞_, area under the concentration-time curve from zero to infinite; AUMC, area under the first moment of the concentration-time; MRT, mean residence time; Vss, apparent volume of distribution at equilibrium determined after intravenous administration; Vz/F, apparent volume of distribution during terminal phase after extravascular administration; F_0-t_, fraction of the administered dose systemically available between time zero and a definite time; F_0-∞_, fraction of the administered dose systemically available between time zero and infinity: F, absolute bioavailability. NA: not applicable; ND: Not determined. The PK parameters were calculated from the data from the three sheep that make up each group. ^C^ Only the value for an individual could be calculated.

## Supplementary Materials

The following are available online at https://www.mdpi.com/2072-6651/12/7/455/s1, Figure S1: Reverse phase HPLC separation of the venom of *C. simus*. Figure S2: Western blot showing that the specific antibodies lack cross recognition against the other protein families. Figure S3: Level of recognition by ELISA of anti-SVMP and anti-SVSP antibodies to SVMPs and SVSPs. Table S1: Quantification of fibrinogen in G2 sheep. Table S2: Quantification of fibrinogen in G3 sheep. Table S3: Quantification of WV, SVMPs, SVSPs and Crotoxin in group 1. Table S4: Quantification of WV, SVMPs, SVSPs and Crotoxin in group 2. Table S5: Quantification of WV, SVMPs, SVSPs and Crotoxin in group 3 lymph. Table S6: Quantification of WV, SVMPs, SVSPs and Crotoxin in group 3 blood.
