# Supplementary material for: Neotropical Rattlesnake (Crotalus simus) Venom Pharmacokinetics in Lymph and Blood Using an Ovine Model
Source: Toxins (Basel). 2020 Jul 17;12(7):455. doi: 10.3390/toxins12070455 (PMC7405010; doi:10.3390/toxins12070455)
Supplement: Supplementary file 1 [file toxins-12-00455-s001.zip › toxins-851695-sp 2/toxins-851695-supplementary_Neri.pdf]

Supplementary Materials: Neotropical Rattlesnake (*Crotalus simus*) Venom Pharmacokinetics in Lymph and Blood Using an Ovine Model

Edgar Neri-Castro, Melisa Bénard-Valle, Dayanira Paniagua, Leslie V. Boyer, Lourival D. Possani, Fernando López-Casillas, Alejandro Olvera, Camilo Romero, Fernando Zamudio and Alejandro Alagón

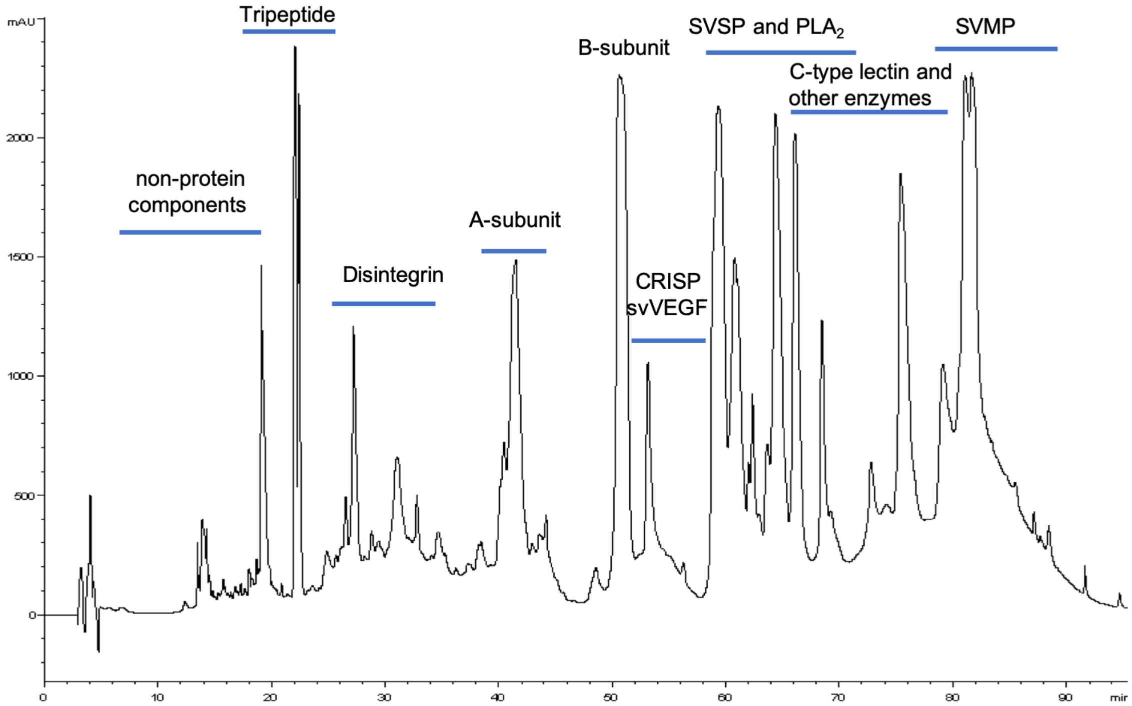

Figure S1. phase HPLC separation of the venom of *C. simus.* The assignment of protein families was made based on the reports of Castro *et al.* (2013) [4] and the results obtained in this study.

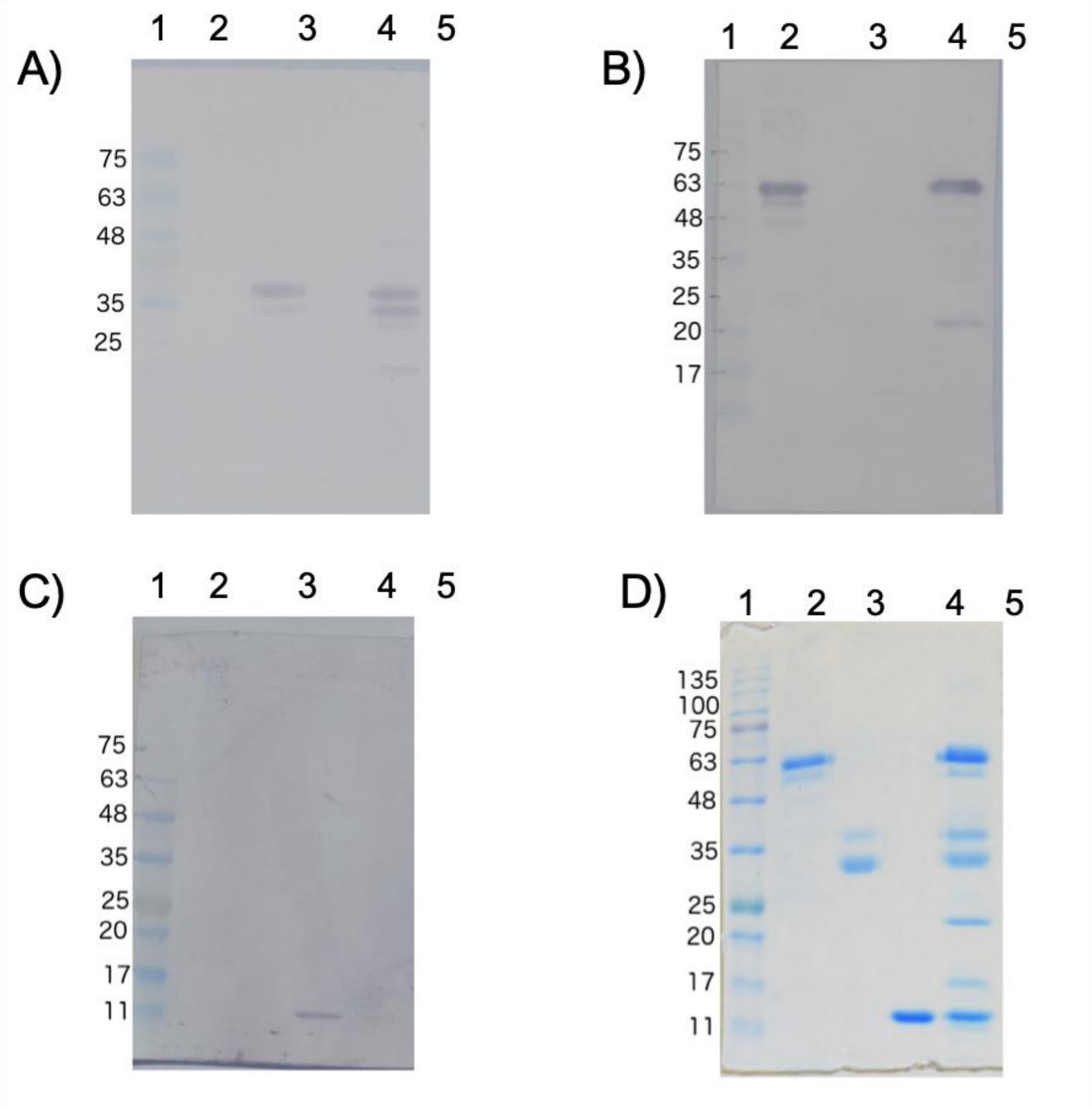

Figure S2. Western blot showing that the specific antibodies lack cross recognition against the other protein families. In each Western blot: lane 1, Standard molecular mass markers; 2, SVMPs; 3, SVSPs; 4, subunit B of crotoxin; 5, WV. For each of the fractions 2 µg per lane were analyzed, while for WV 3 µg were analyzed. (**A**) Shows the specific recognition of anti-SVSP antibodies to purified SVSPs and those in WV; (**B**) Shows the specific recognition of anti-SVMPs antibodies to purified SVMPs and those in WV; (**C**) Shows the specific recognition of monoclonal 4F6 antibody to purified subunit B of crotoxin and those in WV. (**D**) SDS-PAGE 12.5%, in lanes 2 to 4, 2 µg were analyzed and in lane 5, 20 µg.

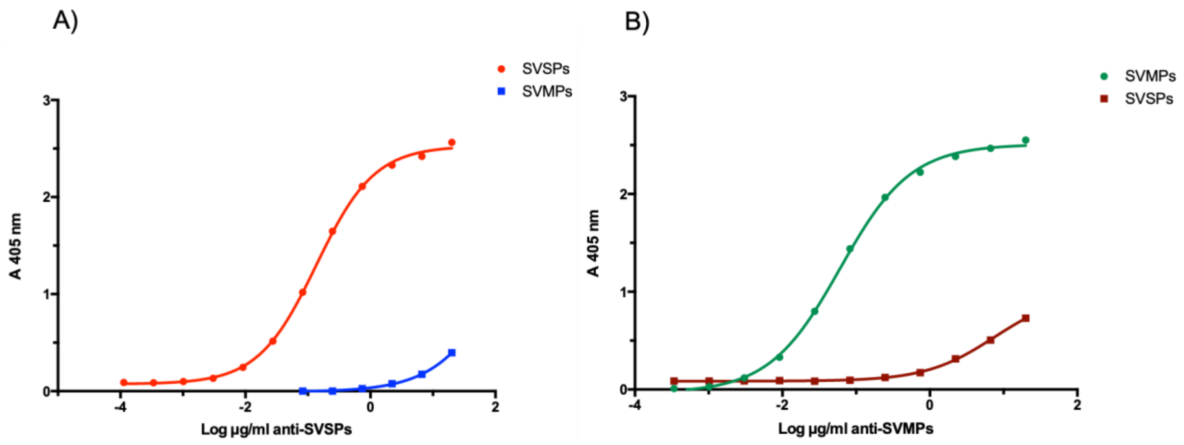

Figure S3. Level of recognition by ELISA of anti-SVSP (**A**) and anti-SVMP (**B**) antibodies to SVMPs and SVSPs.
